# The impact of negative emotions on organizational citizenship behavior and counterproductive work behavior: a self-control perspective

**DOI:** 10.3389/fpsyg.2026.1742931

**Published:** 2026-04-22

**Authors:** Can Liu, Hui Ni, Xin-Tao Meng, Jia Duan

**Affiliations:** 1 Dong Fureng Institute of Economic and Social Development, Wuhan University, Wuhan, China; 2 School of Business, Guangxi University, Nanning, China; 3 School of Economics and Management, Wuhan University, Wuhan, China

**Keywords:** counterproductive work behavior, ego depletion, individual perception, negative emotions, organizational citizenship behavior

## Abstract

**Introduction:**

Negative emotions exert a significant influence on employee behaviors at work.

**Methods:**

Employing Hierarchical Linear Modeling (HLM), we examine the relationships among negative emotions, organizational citizenship behavior (OCB), and counterproductive work behavior (CWB) from the perspective of self-control. Specifically, this study focuses on ego depletion as the key psychological mechanism by which negative emotions affect OCB and CWB. The study also investigates the moderating effects of individual perceptions, specifically perceived organizational support (POS) and perceived coworker support (PCS).

**Results:**

Findings reveal that negative emotions decrease OCB and increase CWB by promoting ego depletion. POS plays a negative moderating role in the relationship between negative emotions and ego depletion, demonstrating a moderated mediating effect on the indirect influence of negative emotions on OCB and CWB via ego depletion. In contrast, the moderating effect of PCS is not significant.

**Discussion:**

This research not only elucidates the theoretical mechanisms underlying the impact of negative emotions on employee behaviors but also offers practical guidance for 2 managers seeking to mitigate the adverse effects of negative emotions and foster a positive organizational environment.

## Introduction

Employee emotions at work refer to the affective states that employees experience during workplace activities (George and Jones, 1996; George and Zhou, 2007). These emotions arise from various events, both personal and work-related (Chi et al., 2013; Diefendorff et al., 2008; Rosado-Solomon et al., 2023). These affective states are generally categorized into positive and negative emotions (George and Zhou, 2007; Tsai et al., 2007), each of which significantly influences employee psychology and physiology. In the actual workplace, these impacts manifest in employees' behaviors, producing both positive and negative effects. For example, positive emotions prompt employees to assist coworkers, respect organizational members, and be concerned about corporate welfare altruistically (Men and Yue, 2019), while workplace loneliness may lead to lower work performance (Ozcelik and Barsade, 2018). With the advancement of organizational behavior research, a growing body of studies has gradually shifted their focus from employees' task performance to their extra-role behaviors (Parke and Seo, 2017). Extra-role behaviors are voluntary actions performed by employees beyond the scope of organizational regulations, which can more fully reflect employees' subjective initiative and thus hold significant research value (Yu et al., 2021).

Organizational Citizenship Behavior (OCB) and Counterproductive Work Behavior (CWB) are two prevalent and highly interrelated types of extra-role behaviors in management practice: the former refers to voluntary behaviors or mutual assistance aimed at promoting organizational performance, while the latter encompasses voluntary behaviors that undermine the interests of the organization or its members. Although OCB is typically seen as promotive and CWB as prohibitive, both are conceptualized as extra-role behaviors because they represent discretionary actions that fall outside the formal requirements of an employee's job description. Extant research has demonstrated that employees‘ emotional states at work influence their cognitive processing and work engagement, which in turn shape their behavioral decisions (Khalid and Syed, 2024). Furthermore, the Affect-Centered Model posits that negative emotions tend to reduce prosocial behaviors and increase harmful ones (Forgas and George, 2001; Lee et al., 2019). Consequently, employees' OCB and CWB are inevitably impacted by negative emotions (Han et al., 2022; Zhang et al., 2018). Scholars hold inconsistent perspectives on the relationship between OCB and CWB (Sackett and DeVore, 2002). Some argue that the two are mutually exclusive, whereas others suggest a significant negative correlation between them. As research has progressed, several studies have found that this negative correlation is not particularly strong; in some cases, there is no relationship or even a positive correlation between the two (Li et al., 2014; Totterdell, 2000). This indicates that the relationship between OCB and CWB is complex and dynamic, thereby highlighting the significance of simultaneously examining the impact of negative emotions on these two types of extra-role behaviors (Spector and Fox, 2002). Furthermore, according to the emotion-centered model of voluntary work behavior, these two behaviors often serve as parallel, emotion-driven responses to the work environment (Spector and Fox, 2002).

Managers often strive to mitigate the negative effects of negative emotions on organizational operations (Eisenberger et al., 2020). However, negative emotions remain challenging to control, as they require strong self-control from employees. Most existing studies have focused on the perspective between individuals. For instance, the behavioral differences between employees who have experienced negative emotions and those who haven't in the workplace. This study, however, attempts to explore from the internal perspective of individuals who have experienced negative emotions. What mechanisms within this individual lead to organizational citizenship behavior and counterproductive behavior? Fishbach and Labroo (2007) argued that personal emotions influence self-control, and some research indicates a link between negative emotions and self-control failure (Leith and Baumeister, 1996), thereby increasing the likelihood of destructive behaviors. Hurley (2023) conceptualized self-control as a limited resource. As individuals constantly exert self-control, their self-control resources will be depleted, leading to individual ego depletion, much like muscles becoming tired from exercise (Troll et al., 2023). Ego depletion represents a state where limited psychological resources are exhausted, which simultaneously affects cognition and behavior. Cognitively, it leads to a reduction in the ability to process information deeply and sustain focus; behaviorally, this cognitive shift manifests as a failure to override impulsive urges, which reduces prosocial behaviors (Dewall et al., 2008) and increases unethical actions (Gino et al., 2011; Zor et al., 2022). Therefore, ego depletion is proposed as an important mediator in this study. While negative emotions provide the affective impulse, managing these emotions in the workplace requires significant self-regulation, which rapidly consumes limited psychological resources. Ego depletion bridges the gap between feeling and doing; it explains that negative emotions not only make people want to act out, but they actively drain the self-control resources needed to maintain professional decorum. Thus, examining how negative emotions influence ego depletion can further reveal their impact on employee behaviors.

Although prior research has examined the effects of negative emotions, there is still a limited understanding of how individual perceptions might alleviate or intensify these impacts. Drawing on existing research (Duke et al., 2009), this study intentionally selected Perceived Organizational Support (POS) and Perceived Coworker Support (PCS) as moderator variables instead of general perceived organizational support. The core rationale is that POS and PCS are more targeted, focusing on the key “employee-organization” and “employee-coworker” exchange dimensions primary sources of non-material support for employees and core factors influencing their emotions and behaviors in organizational settings. Based on Social Exchange Theory, this study incorporates POS and PCS into the research framework to explore their moderating roles in the relationships among negative emotions, ego depletion, and employee behaviors (OCB, CWB)-a logic highly aligned with the theory's core (Eisenberger et al., 1986). Theoretically, POS and PCS are two key non-material exchange resources for employees: POS reflects perceptions of organizational care, respect, and support (Reynolds-Kueny and Shoss, 2021). High POS fosters a reciprocal tendency to repay the organization, buffering negative emotions and ego depletion, reducing CWB, and increasing OCB. PCS reflects perceptions of coworkers' willingness to provide work and emotional support; such supportive exchanges create a positive interpersonal climate, alleviate negative emotions, offset ego depletion-related resource loss, and thus moderate core variable relationships (Dewall et al., 2008).

From the perspective of self-control, this study collects data on employees' emotions and behaviors through questionnaires. Based on these data, it examines the impact of negative emotions on OCB and CWB through ego depletion. Additionally, the present study considers individual perceptions (POS and PCS) as moderating variables and employs Hierarchical Linear Modeling (HLM) as the research tool. This cross-level design aims to comprehensively examine the influences of relevant factors at both the within-individual and between-individual levels.

### Theoretical framework and hypothesis development

As a core theory explaining individual emotion regulation and behavioral decision-making, Self-Control Theory posits that individuals' self-control ability stems from limited psychological resources. When individuals experience negative emotions, their self-control resources are prone to depletion (i.e., ego depletion), which in turn affects their subsequent work attitudes and behavioral performance. Social Exchange Theory (SET), by contrast, focuses on reciprocal interactions at the interpersonal and organizational levels, arguing that individuals' behavioral decisions in organizational contexts are based on cost-benefit trade-offs and the norm of reciprocity. Perceived Organizational Support (POS) and Perceived Coworker Support (PCS), as important non-material exchange resources perceived by employees, influence their judgments of the relationship between emotional experiences and behavioral outcomes.

### The impact of negative emotions on employee behaviors

Negative emotions refer to affective states triggered by internal or external factors in specific situations. These states, such as sadness, anger, tension, pain, fear, and hatred, are unfavorable to maintaining continuous work or normal thinking. Research shows that employees' negative emotions at work negatively affect task performance (van Steenbergen et al., 2021) and lead to lower work outcomes (Rothbard and Wilk, 2011). Additionally, negative emotions have a significant impact on employees' attitudes and thoughts (Han et al., 2022). Sackett and DeVore (2002) categorized employees' behavior in the workplace into organizational citizenship behavior (OCB) and counterproductive work behavior (CWB). Spector and Fox (2002), using the emotion-centered model, showed that negative emotions tend to increase employees' CWB and decrease their OCB.

OCB refers to employees' discretionary behaviors that are not directly recognized by the formal reward system. These contributory behaviors include helping coworkers meet deadlines, protecting the organization's image, and assisting new employees to feel at home (Lee and Allen, 2002). As an extra-role behavior, OCB is voluntary for employees and thus is susceptible to emotional influences (Connelly et al., 2022). Meanwhile, the mood congruence theory posits that individuals tend to acquire information in line with their current mood and exhibit corresponding behaviors (Hou and Cai, 2022). Therefore, employees' OCB levels may be affected by negative emotions. This is because when employees experience negative emotions, they tend to perceive and evaluate the work environment and events in a detrimental manner (Rothbard and Wilk, 2011), which may further lead them to form negative evaluations of the organization and its coworkers. And finally, these negative evaluations may reduce employees' motivation to voluntarily devote themselves to the organization and coworkers (Lee and Allen, 2002). Additionally, when negative emotions are inconsistent with job requirements, employees must suppress their negative emotions. This suppression leads to emotional exhaustion, and employees experiencing emotional exhaustion are less likely to engage in OCB (Trougakos et al., 2015). In contrast, employees' positive emotions are associated with more prosocial behaviors (Michie, 2009; van Kleef and Lelieveld, 2022), which also implies that negative emotions may reduce employees' OCB. Thus, we propose H1:

H1: Employees' negative emotions have a negative impact on OCB.

CWB refers to deliberate behaviors exhibited by employees that violate organizational norms and threaten the well-being of the organization and its members (Mackey et al., 2021). These detrimental behaviors include engaging in personal matters during work, treating coworkers with a hostile attitude, mocking and verbally attacking others, all of which can cause harm to the organization or its stakeholders. Some scholars argue that CWB is employees' negative response to work (Sharma and Chillakuri, 2023). According to the mood congruence theory, negative emotions may lead employees to form negative evaluations of the work environment, which in turn cause them to exhibit more CWB (Hou and Cai, 2022). For instance, when employees experience negative emotions, they are more inclined to engage in CWB (Samnani et al., 2014; Yang and Diefendorff, 2009) and deviant behaviors (Zhang et al., 2018). Additionally, negative emotions can increase the possibility of impulsive (Denson et al., 2011) and aggressive behaviors (Watkins et al., 2015). These studies demonstrate that negative emotions represent a detrimental psychological state, indicating that the self has been harmed or threatened. However, Liu et al. (2022) argued that such negative emotions can be mitigated through external venting. From this perspective, CWB can help satisfy employees‘ need for venting, thereby contributing to emotional recovery (Koopman et al., 2021; Miner and Glomb, 2010). Therefore, when employees experience negative emotions, they may be more likely to engage in CWB as a means to vent their negative feelings. In summary, the H2 is proposed:

H2: Employees' negative emotions have a positive impact on CWB.

#### The mediating role of ego depletion

The concept of self-control resource depletion derives from Baumeister et al.'s limited self-control model, which posits that: (1) Individuals consume self-control resources during self-control, and such resources are short-term limited; (2) An individual's self-control ability is closely linked to their resource availability—the more sufficient the resources, the better their performance on self-control-related tasks; (3) All self-control behaviors consume the same resources; even across different domains, prior self-control behaviors reduce subsequent self-control ability. Conscious self-control continuously depletes resources; when depletion reaches a threshold, individuals enter a state of weak self-control, namely self-control resource depletion.

Self-control refers to the ability to intentionally override, modify, or suppress immediate, satisfying behaviors in pursuit of long-term goals (Dewall et al., 2008). Troll et al. (2023) conceptualized the self-control ability as a limited resource, emphasizing that constant daily activities can deplete this resource. For example, employees must maximize the use of their self-control resources to avoid cognitive distraction, align their behaviors with social expectations, and manage work demands such as time pressure and task conflict (Vahle-Hinz et al., 2021; Dai et al., 2015; Lanaj et al., 2014; Lian et al., 2014). All these activities can lead to depletion of self-control resources, also known as ego depletion (Dewall et al., 2008).

Employees‘ negative emotions may lead to ego-depletion. Negative emotions can adversely affect employees' task performance and work communication, requiring them to invest effort and energy to address these issues (Vohs et al., 2005; Vohs and Faber, 2007). For example, negative emotions lead to employees becoming more aggressive and less friendly during the actual work process (Watkins et al., 2015). However, such unfriendliness is not always an intentional behavior on the part of employees, and it may cause obstacles to their work. To avoid such situations, employees must learn to control and regulate these negative emotions and treat others with a more positive attitude. Therefore, the management of negative emotions demands a higher level of self-control and leads to ego depletion (Diestel et al., 2015), particularly when employees are required to suppress their genuine emotions and display inauthentic affective states (Zapf and Holz, 2006). This suggests that negative emotions can contribute to ego depletion. As a result, we propose H3:

H3: Negative emotions have a positive impact on ego depletion.

OCB does not emerge automatically (Dewall et al., 2008), it requires the self-sacrifice of employees. For example, when employees help coworkers meet deadlines, they often have to sacrifice their own rest time. Therefore, Dewall et al. (2008) proposed that, compared with daily in-role behaviors, OCB requires employees to possess more significant self-control resources to resolve the motivational conflict between helping others and being self-centered. Furthermore, Lian and Brown (2014) demonstrated that self-control can effectively reduce employees' hostility toward leaders, even when leaders engage in inappropriate management. This suggests that employees with high-level self-control are more likely to exhibit OCB. At the same time, individuals with great self-control are more likely to achieve success in interpersonal interactions (Tangney et al., 2004), and such interpersonal success also has a positive impact on employees' engagement in OCB. Additionally, research has confirmed that participants in a state of ego depletion engage in fewer prosocial behaviors than those in a non-depleted state (Dewall et al., 2008). This is because when employees are in a state of ego depletion, they develop the desire to conserve limited resources, thereby reducing their willingness to engage in prosocial behaviors (Hobfoll, 1989). Therefore, negative emotions lead to greater ego depletion, while greater ego depletion makes it difficult for employees to overcome self-centered motivational conflicts, hinders them from achieving success in interpersonal interactions, and leads to their desire to conserve self-control resources, all of which result in less OCB. In conclusion, the H4 is proposed:

H4: ego depletion mediates the impact of negative emotions and OCB.

CWB, as a kind of behavior that exerts adverse impacts on the organization and coworkers, is influenced by self-control. Loewenstein (1996) proposed that the lack of individuals' self-control not only inhibits their tendency to engage in prosocial behaviors but also makes them more likely to succumb to impulses that satisfy short-term desires. In addition, Ghoniem and Hofmann (2021) demonstrated that individuals who experience self-control failures are more likely to engage in irrational consumption and impulsive purchasing behaviors. Specifically, those with ego depletion are more likely to consume unhealthy food, use drugs, and abuse alcohol (Rafael and Lopes, 2023). Some scholars argue that employees in a state of ego depletion tend to violate organizational contracts and engage in opportunistic behaviors (Reason et al., 1998). For example, participants who have undergone ego depletion often violate moral principles and yield to short-term temptations (Gunia, 2022). Individuals who experience ego depletion exhibit more dishonest behaviors (Mead et al., 2009) and unethical behaviors (Gino et al., 2011). Additionally, self-control resources are limited and decrease with the impact of relevant activities, indicating that self-control resources require a certain amount of time to recover. For instance, insufficient sleep can promote self-regulatory resource depletion, which in turn affects employees' work engagement (Lanaj et al., 2014) and increases the likelihood of deviant behaviors at work (Christian and Ellis, 2011). Therefore, negative emotions can lead to greater ego depletion, which in turn results in more CWB. In summary, the H5 is proposed:

H5: Ego depletion mediates the impact of negative emotions and CWB.

### The moderating roles of POS and PCS

Employees can perceive both support from the organization and coworkers during work, and differences in organizational environment and contexts lead to variations in these two types of perceptions among different individuals. Although individual perceptions represent individuals' subjective cognitions and judgments, they are not fabricated out of thin air. Instead, they are reflections of objective realities. According to social exchange theory, the support that employees perceive from the organization and from coworkers has an influence on their psychological and behavioral at work (Eisenberger et al., 1986).

Perceived organizational support (POS) refers to the extent to which employees perceive their organization as supportive (Eisenberger et al., 1986). This support may come from tangible resources (e.g., salary) or intangible resources (e.g., respect) (Eisenberger et al., 2004). Based on social exchange theory, employees who receive more support from enterprises can acquire additional resources, enabling them to better cope with external pressures (Eisenberger et al., 1986). For example, the POS can mitigate the negative impact of customer mistreatment on employees (Wang et al., 2013), alleviate the negative effects of high workload on employees' daily well-being (Ilies et al., 2010), enables expatriates to adapt more quickly to the pressures of international assignments (Takeuchi et al., 2009), and reduces the impact of coworkers' withdrawal behavior on employees' own withdrawal (Eder and Eisenberger, 2008). Based on the above studies, employees with higher POS are more likely to develop positive emotions and attitudes toward work and the organization, which helps them effectively cope with negative emotions. This is conducive to mitigating the impact of negative emotions on ego depletion. In addition, enterprises that provide higher POS may allow employees to obtain more rest time. This not only alleviates the impact of negative emotions on ego depletion but also helps employees recover their self-control resources. Thus, we proposed H6:

H6: POS plays a negative moderating role in the impact of negative emotions on ego depletion. Specifically, the higher the POS, the weaker the negative emotions' impact on ego depletion.

Perceived coworker support (PCS) refers to the perceived support that employees receive from their coworkers, as well as the extent to which coworkers provide assistance (Xu and Yang, 2021). It plays a significant role in employees' cognitive processes. On the one hand, PCS can influence employees' emotional exhaustion and psychological depletion. The existing study demonstrates that when employees experience mistreatment at work, higher PCS can mitigate the negative impact of such unfair treatment (Sloan, 2016). Moreover, PCS can help reduce the impact of abusive supervision on employees' emotional exhaustion (Wu and Hu, 2009). When employees receive sufficient support from coworkers, work mistreatment does not necessarily lead to depletion or negative work behaviors and attitudes (Xu and Yang, 2021). On the other hand, when employees with higher PCS experience negative emotions, they can alleviate these emotions through communication with coworkers or by receiving support from them, thereby mitigating ego depletion. Fisher (1985) argued that support from coworkers enables employees to feel that they are in a suitable environment, promoting psychological well-being, which helps reduce the impact of external activities on limited self-control resources. Additionally, PCS can help employees improve work efficiency and complete tasks more quickly. In this way, employees can obtain more time for rest and adjustment, which is further conducive to the recovery of self-control resources. As a result, we propose H7:

H7: PCS plays a negative moderating role in the impact of negative emotions on ego depletion. Specifically, the higher the PCS, the weaker the negative emotions' impact on ego depletion.

### The moderated mediating effect

We have reorganized and elaborated on the moderated mediation proposal process. Based on Social Exchange Theory (SET), individuals' attitudes, behaviors, and psychological cognition in organizational contexts are essentially adaptive responses to evaluated social exchange resources (Eisenberger et al., 1986). Specifically, varying levels of Perceived Organizational Support (POS) and Perceived Coworker Support (PCS) lead to significant differences in employees' work attitudes (e.g., job satisfaction, organizational commitment), behaviors (e.g., in-role, extra-role behavior), and psychological states (e.g., self-efficacy, psychological safety). These differences exert a heterogeneous moderating effect on the indirect path “negative emotions → self-control resource depletion → Organizational Citizenship Behavior (OCB)/Counterproductive Work Behavior (CWB),” forming a moderated mediation mechanism.

Regarding POS's moderating role, high POS effectively enhances employees' psychological capital (Luthans et al., 2004). Luthans (2004) defines psychological capital as a developable positive psychological state encompassing confidence (self-efficacy), hope, optimism, and resilience—traits that form a psychological buffer against negative emotions and resource depletion. Day et al. (2021) further confirm that high-quality interpersonal relationships and high POS improve individuals' positive psychological and physical states, strengthening emotional regulation and resource recovery through internal potential activation, thereby reducing self-control resource consumption from negative emotions.

For PCS's moderating role, high PCS reduces employees' psychological contract breach and work stress (van Steenbergen et al., 2021). Coworker supportive interactions provide emotional comfort and practical help, alleviating negative emotions from stress or conflicts and reducing ineffective psychological resource consumption. Luthans et al. (2004) notes that leaders' resilience positively influences employees via demonstration effects, which is strengthened in high-PCS teams, indirectly enhancing psychological stability. Overall, high POS and PCS together stabilize employees' psychological states, improve tolerance to negative emotions, and minimize interference with the self-control resource system.

Wehrt et al. (2022) support that emotional stability weakens negative emotions' effects and reduces self-control resource depletion. Thus, when negative emotions indirectly impact OCB and CWB via increased self-control resource depletion, the stable psychological state shaped by high POS and PCS enhances self-control ability. Specifically, it improves negative emotion identification and regulation efficiency, reducing impulsive decisions and resource waste, thereby alleviating the indirect path's intensity and mitigating negative behavioral impacts.

High POS and PCS also provide substantial help and emotional encouragement to reduce external negative impacts. High POS offers reasonable work support, clear role guidance, and fair incentives, reducing negative emotions and resource depletion from organizational factors (e.g., ambiguous tasks, unfair incentives). High PCS enables coworker collaboration and experience sharing, lowering task-related pressure and negative emotions from failure or conflicts. This “low external impact” state reduces self-control resource depletion, weakening negative emotions' indirect effects on OCB and CWB at the source and ultimately moderating the entire mediation process. In summary, the following hypotheses are proposed:

H8a: POS moderates the indirect effect of negative emotions on OCB through ego depletion. Specifically, this indirect effect is weaker when POS is higher.

H8b: POS moderates the indirect effect of negative emotions on CWB through ego depletion. Specifically, this indirect effect is weaker when POS is higher.

H9a: PCS moderates the indirect effect of negative emotions on OCB through ego depletion. Specifically, this indirect effect is weaker when PCS is higher.

H9b: PCS moderates the indirect effect of negative emotions on CWB through ego depletion. Specifically, this indirect effect is weaker when PCS is higher.

**Figure 1:**
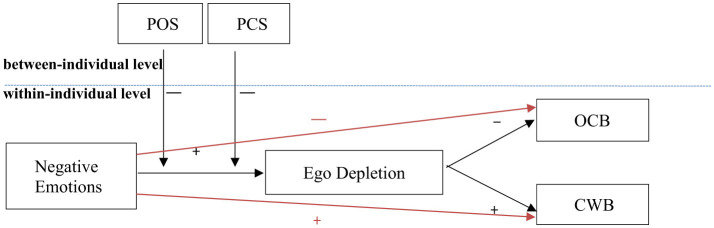
The theoretical model.

The overall theoretical model of the present study is illustrated in Figure 1.

## Methodology

### Sample and procedures^1^

The data for the present study were collected through a questionnaire survey involving 155 employees from nine companies in Wuhan. Most items in the questionnaire adopted a 7-point Likert scale. The questionnaire was divided into two parts: The first part was completed by respondents on the morning of the first workday or in advance, and it was used to collect data on between-individual level variables (Level 2 variables). The second part consisted of a diary work booklet covering 10 workdays. Respondents were required to complete it within 5–10 min, either before leaving work or after returning home each day. The content to be filled out was identical every day, aiming to record respondents' different daily emotions and collect data on within-individual level variables (Level 1 variables).

The selection of employees from enterprises in Wuhan as research subjects was mainly based on the alignment between the research theme and local enterprise management practices. Additionally, the sample basically covered the core job types of small and medium-sized enterprises, which could meet the basic requirements for testing the core hypotheses of this study. Although Hierarchical Linear Modeling (HLM) analysis is more suitable for large-scale cross-level samples, this study minimized the impact of the small sample size on the reliability of the analysis results by strictly controlling intra-level errors and optimizing model fit.

A total of 196 questionnaires were distributed, and 155 valid questionnaires were recovered, resulting in a response rate of 79.1%. Among the 155 respondents, 40.6% were administrative staff, 28.9% were marketing and sales, 21.2% were research and development staff, and 10.3% were production and operation staff. The gender distribution included 51.6% females and 48.4% males. The vast majority of participants (97.4%) had received a college education. The participants' average weekly working hours were 43.6 h.

This study employed the work diary method for data collection primarily because this method enables the capture of dynamic changes in employees' daily emotions and behaviors. By doing so, the collected data can align with employees' actual work experiences, which facilitates a more robust examination of the relationship between negative emotions and employee behaviors. Furthermore, the selection of a 10-workday (i.e., 2-week) period as the survey cycle is supported by objective evidence. First, this duration is consistent with the existing literature on diary studies (Jiang et al., 2024). Second, it conforms to the recommendations proposed by Wheeler and Reis (1991), who argued that a 2-week timeframe is sufficient to capture the fluctuating patterns in participants' work experiences.

### Research variables and measurements

#### Variables

The data in this study are organized into two levels. The first level is the within-individual level, encompassing variables that fluctuate with employees' daily work experiences, including negative emotions, ego depletion, OCB, and CWB. The second level is the between-individual level, including variables that are relatively stable across individuals, covering POS and PCS. The connotations of the variables involved in this study are presented in Table 1.

**Table 1:** Variables description.

Level	Variables description		Variables symbol
Within-individual level	Control variables	Dummy variable for weather 1	weather1
		Dummy variable for weather 2	weather2
		Outdoor work hours	PE
	Dependent variable	Negative emotions	NM
	Independent variable	Organizational citizenship behavior	OCB
		Counterproductive work behavior	CWB
	Mediator variable	Ego depletion	ED
Betwenn-individual level	Moderator variable	Perceived organization support	POS
		Perceived coworker support	PCS

#### Measurements

All scales used in this study have been culturally adapted for Chinese employees and validated in the Chinese context.

Negative emotions were measured using the Positive Affect and Negative Affect Scale (PANAS), developed by Watson et al. (1988). This scale includes eight items, such as “Interested,” “Distressed,” “Excited,” and “Upset.” The Cronbach's α coefficient of the scale was 0.948. The participants were not required to complete a positive emotion measure.

#### Ego depletion

Ego depletion was measured using a scale from the self-control literature, developed by Lanaj et al. (2014), which included five items such as “I feel drained,” “My mind feels unfocused right now,” “ Right now, it would take a lot of effort for me to concentrate on something,” “My mental energy is running low,” and “I feel like my willpower is gone.” The Cronbach's α coefficient of the scale was 0.951.

#### Organizational citizenship behavior

OCB was measured using five items developed by Lee and Allen (2002), including items “Express loyalty toward the organization,” “Take action to protect the organization from potential problems,” and “Demonstrate concern about the image of the organization.” The Cronbach's α coefficient of the scale was 0.816.

#### Counterproductive Work Behavior

CWB was measured using five items developed by Mackey et al. (2021), including items “Work on your own matters instead of working for organization,” “Spend too much time fantasizing or daydreaming instead of working,” and “Make fun of someone at work.” The Cronbach's α coefficient of the scale was 0.806.

#### Perceived organizational support

POS was measured using an eight-item scale developed by Lynch et al. (1999). Sample items include “My organization strongly considers my goals and values,” “My organization really cares about my well-being,” and “Help is available from my organization when I have a problem.” The Cronbach's α coefficient of the scale was 0.752.

#### Perceived coworker support

PCS was measured using a six-item scale developed by Morgeson and Humphrey (2006). Sample items include “I have the opportunity to develop close friendships in my job,” “ People I work with are friendly,” and “ I have the chance in my job to get to know other people.” The Cronbach's α coefficient of the scale was 0.862.

#### Control variable

Two control variables were selected at the within-individual level of the present study: daily weather and employees' daily outdoor working hours, both of which were recorded in the work diaries. Weather was chosen as a control variable because the present study focuses on exploring the impact of negative emotions on employees' behaviors, and weather is a well-documented factor that influences individuals' emotional states (Bellet et al., 2023). Employees' outdoor working hours were measured directly in hours. This variable was selected as a control variable because the outdoor work environment is more likely to impose shocks on employees' self-control resources, for example, exposure to air pollution (Lanaj et al., 2014).

### Data analysis

Given that the study's data are structured into two levels (within-individual and between-individual), SPSS was used as the tool for data management, and hierarchical linear modeling (HLM) was employed as the analytical tool to test the proposed hypotheses. Using HLM 6.08, we conducted group-mean centralization on the variables of the within-individual level to eliminate the influence of variance among individuals on these variables, and carried out grand-mean centralization on the variables of the between-individual level. Regarding mediating effects, the steps proposed by Ge (2023) were adopted for testing. After verifying the mediating effects, the moderating effects were tested by examining whether the correlation coefficients of the interaction term between the moderating variables and the independent variable were significant. Finally, R software was used to test the moderated mediating effects, with the key criterion being whether 0 was included in the confidence interval.

## Results

### Descriptive statistics and correlation coefficients

Table 2 presents the descriptive statistics of the main variables in this study, including the mean and standard deviation of each variable, as well as the correlation coefficients among the within-individual level variables. As shown in the table, the standard deviations of negative emotions, ego depletion, OCB, and CWB are relatively large, with noticeable differences. This indicates that employees‘ emotions and behaviors at work exhibit significant fluctuations. Additionally, the mean value of OCB is relatively high, indicating that employees tend to engage in more extra-role behaviors at work and possess a strong sense of responsibility toward the organization. Furthermore, negative emotions are significantly negatively correlated with OCB and significantly positively correlated with CWB. Meanwhile, negative emotions are significantly positively correlated with ego depletion. Ego depletion is significantly negatively correlated with OCB and positively correlated with CWB. Regarding the control variables, it can be observed that outdoor working hours are positively correlated with ego depletion, suggesting that the outdoor environment and activities consume the limited resources of self-control. Weather has a significant impact on CWB, implying that the quality of weather is significantly related to employees' bad behaviors (i.e., CWB), which is consistent with management practices.

**Table 2:** Descriptive statistics of variables.

Variables	Mean	Standard deviation	1	2	3	4	5	6	7
Within-individual level									
1. weather1	0.58	0.49	1						
2. weather2	0.27	0.44	−0.713^**^	1					
3. PE	2.65	1.76	−0.011	0.001	1				
4. NM	2.03	1.04	0.012	0.039	−0.050^*^	1			
5. ED	2.64	1.3	−0.069^**^	0.059^*^	0.071^**^	0.613^**^	1		
6. OCB	4.67	1.11	−0.028	−0.012	0.001	−0.187^**^	−0.260^**^	1	
7. CWB	2.74	0.97	−0.087^**^	0.019^**^	0.047	0.369^**^	0.425^**^	−0.244^**^	1
Between-individual level									
8. POS	4.41	0.75							
9. PCS	3.88	0.56							

^*^p < 0.05.

^**^p < 0.01.

For the between-individual level moderating variables, both POS and PCS have relatively high mean values and minor standard deviations. This indicates that employees generally perceive a high level of POS and PCS, and with relatively slight variations. Additionally, the mean value of POS is higher than that of PCS, indicating that employees perceive more support from the organization, and POS plays a more significant role in employees' psychological cognition.

### Analysis of variance components

Before testing the hypotheses proposed in this study, it is first necessary to conduct a variance component analysis on the within-individual level variables. The purpose of this analysis is to verify whether these variables exhibit meaningful variations within individuals. Here, the null model in HLM was used to perform the variance component analysis, and the results are presented in Table 3.

**Table 3:** Variance component analysis.

Variables	Intercept	Within-individual variance	Between-individual variance	Percentage of variance within individuals
Negative emotions	2.04^***^	0.34	0.74^***^	31%
Ego depletion	2.64^***^	0.66	1.04^***^	39%
Organizational citizenship behavior	4.67^***^	0.46	0.77^***^	37%
Counterproductive work behavior	2.74^***^	0.37	0.58^***^	31%

^***^p < 0.001.

As shown in Table 3, 31% of the total variance in negative emotions is attributed to within-individual variance (intra-group variance); 39% of the total variance in ego depletion is attributed to within-individual variance; 37% of the total variance in OCB is attributed to within-individual variance; 31% of the total variance in CWB is attributed to within-individual variance.

These data support the study's premise that there are meaningful differences both within and between individuals. Moreover, they confirm the validity of proceeding with the subsequent cross-level analysis.

### Common method variance analysis

Since all variable data in this study were self-assessed by employees, respondents may have different levels of understanding of the same item, leading to the problem of common method variance (CMV)—i.e., systematic variance caused by the measurement method—which further affects the explanatory power among variables. Therefore, Harman's single-factor test was conducted on the data. Factor analysis of all variables showed no single factor emerged; the first principal component explained 27.68% of the variance, which is less than half of the total explained variance (71.33%), indicating that there is no common method variance problem.

### Convergent validity and discriminant validity

Confirmatory Factor Analysis (CFA) can be used to test the fitness of the theoretical model, and it can also be employed to test the convergent validity and discriminant validity of the scale through the obtained Average Variance Extracted (AVE) values. Since the theoretical model of this study includes variables at two levels with different sample sizes (155 at the within-individual level and 1,550 at the between-individual level), this study refers to the practices of similar studies and conducts CFA for the variables at the first level. The results of CFA obtained using AMOS software are presented in the table. In addition to testing the four-factor model, other factor models were also verified: the three-factor model was formed by merging two variables, namely negative emotions and ego depletion; there were two two-factor models, one formed by merging negative emotions and ego depletion, and organizational citizenship behavior (OCB) and counterproductive work behavior (CWB) respectively, and the other formed by merging negative emotions, ego depletion, and CWB; the single-factor model was formed by merging all four variables. As shown in the Table 4, compared with the single-factor, two-factor, and three-factor models, the four-factor model achieved the most ideal fitting effect with the actual data (/df = 14.179, CFI = 0.957, NFI = 0.954, IFI = 0.936, TLI = 0.957, RMSEA = 0.088).

**Table 4:** Convergent validity and discriminant validity analysis.

Model	χ^2^/df	NFI	IFI	TLI	CFI	RMSEA
Four factors (OCB, CWB, ED, NM)	14.179	0.954	0.936	0.957	0.957	0.088
Three factors (OCB, CWB, ED + NM)	72.774	0.748	0.673	0.676	0.750	0.206
Two factors (OCB + CWB, ED + NM)	102.96	0.629	0.631	0.540	0.631	0.245
Two factors (OCB, CWB + ED + NM)	97.682	0.648	0.650	0.564	0.650	0.239
One factors (OCB + CWB + ED + NM)	127.384	0.532	0.534	0.430	0.534	0.273

When conducting Confirmatory Factor Analysis (CFA) of the scale, the obtained Average Variance Extracted (AVE) values can be used to measure the convergent validity and discriminant validity of the scale. Since each variable has one AVE value, and the verification of discriminant validity requires comparing the square root of the AVE value with the correlation coefficients between variables, this study presents the AVE values in the table displaying the results of the correlation analysis between variables. As shown in the table, the AVE value of negative emotions is 0.75, the AVE value of ego depletion is 0.84, the AVE value of Organizational Citizenship Behavior (OCB) is 0.62, the AVE value of Counterproductive Work Behavior (CWB) is 0.61, the AVE value of Perceived Organizational Support (POS) is 0.58, and the AVE value of Perceived Colleague Support (PCS) is 0.61. These values range from 0.58 to 0.84, all of which are greater than the critical value of 0.5, indicating that the measurement scales of the variables in this study all have good convergent validity. The square root of the AVE value of each variable in this study is greater than the correlation coefficient between that variable and other variables, which meets the requirements for testing discriminant validity. This indicates that the variables in the theoretical model of this study have good discriminant validity and that the variables indeed belong to six different constructs, which also confirms the feasibility of subsequent analyses.

### Hypothesis testing

#### The within-individual level

H1 posits that negative emotions have a negative impact on OCB. As shown in Model 2 of Table 5, after controlling for the control variables, negative emotions exhibit a significant negative effect on OCB (*r* = −0.10, *p* < 0.05). Therefore, H1 is supported. H2 proposes that negative emotions have a positive impact on CWB. From Model 5 of Table 5, it can be observed that, after controlling for the control variables, negative emotions have a significant positive effect on CWB (*r* = 0.15, *p* < 0.01). Thus, H2 is supported. H3 suggests that negative emotions have a positive impact on ego depletion. As indicated in Model 8 of Table 5, after controlling for the control variables, negative emotions have a significant positive effect on ego depletion (*r* = 0.48, *p* < 0.01). Consequently, H3 is supported.

**Table 5:** HLM analysis results.

Variable	OCB			CWB			ED					
	Mode 1	Mode 2	Model 3	Mode 4	Model 5	Model 6	Model 7	Model 8	Model 9	Model 10	Model 11	Model12
Intercept	4.67^**^	4.67^**^	4.67^**^	2.74^**^	2.74^**^	2.74^**^	2.64^**^	2.64^**^	2.64^**^	2.64^**^	2.64^**^	2.64^**^
Control variables												
weather1	−0.12	−0.11	−0.11	0.03	0.01	0.01	0.01	−0.05	−0.05	−0.05	−0.10	−0.10
weather2	−0.08	−0.06	−0.07	0.09	0.07	0.07	0.04	−0.03	−0.03	−0.03	−0.06	−0.06
PWH	0.00	0.00	0.00	0.03	0.02	0.02	0.04	0.03	0.03	0.03	0.03	0.03
Independent variable												
NM		−0.10^*^	−0.05		0.15^**^	0.11^*^		0.48^**^	0.48^**^	0.48^**^	0.43^**^	0.43^**^
Mediator variable												
ED			−0.12^**^			0.10^**^						
Moderator variables												
POS									−0.23^**^		−0.23^**^	
PCS										−0.38^**^		−0.38^**^
Interaction term												
NM ^*^ POS											−0.17^**^	
NM ^*^ PCS												−0.04

^*^p < 0.05.

^**^p < 0.01.

In terms of mediating effect testing, H4 posits that ego depletion plays a mediating role between negative emotions and OCB, while H5 proposes that ego depletion exerts a mediating role between negative emotions and CWB. First, regarding the verification of H4, H1 has already confirmed a negative correlation between negative emotions and OCB, and H3 has verified a positive correlation between negative emotions and ego depletion. As shown in Model 3 of Table 5, after incorporating ego depletion into the theoretical model of the impact of negative emotions on OCB, ego depletion is found to have a significant negative effect on OCB (*r* = –0.12, *p* < 0.01). Meanwhile, the relationship between negative emotions and OCB becomes non-significant (*r* = –0.05, *p* > 0.05). In summary, ego depletion plays a complete mediating role in the relationship between negative emotions and OCB. Therefore, H4 is supported. Second, for the verification of H5, H2 has confirmed a positive correlation between negative emotions and CWB, and H3 has verified a positive correlation between negative emotions and ego depletion. As indicated in Model 6 of Table 5, after introducing ego depletion into the theoretical model of the impact of negative emotions on CWB, ego depletion has a significant positive effect on CWB (*r* = 0.10, *p* < 0.01). At the same time, the positive correlation between negative emotions and CWB remains significant (*r* = 0.11, *p* < 0.05), but its correlation coefficient decreases from 0.15 to 0.11. To summarize, ego depletion partially mediates the relationship between negative emotions and CWB. Thus, H5 is supported.

#### The between-individual level

**Figure 2:**
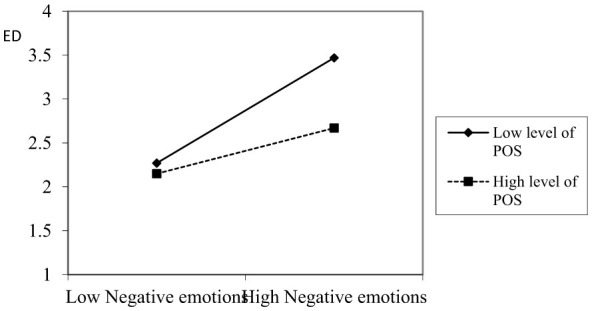
The moderating effect of POS.

H6 posits that POS exerts a negative moderating effect on the relationship between negative emotions and ego depletion. The verification process is as follows: First, as shown in Model 8 of Table 5, there is a significant positive correlation between negative emotions and ego depletion (*r* = 0.48, *p* < 0.01). Second, Model 9 of Table 5 indicates that the main effect of POS on ego depletion is a significant negative correlation (*r* = –0.23, *p* < 0.01). Finally, the results from Model 11 in Table 5 indicate that as POS increases, the positive effect of negative emotions on ego depletion becomes weaker (*r* = –0.17, *p* < 0.01). Together, these findings confirm that POS plays a negative moderating role in the relationship between negative emotions and ego depletion. Therefore, H6 is supported. The moderating effect of POS is illustrated in Figure 2. When POS is low, the positive relationship between negative emotions and ego depletion is more significant. This observation further verifies H6.

**Figure 3:**
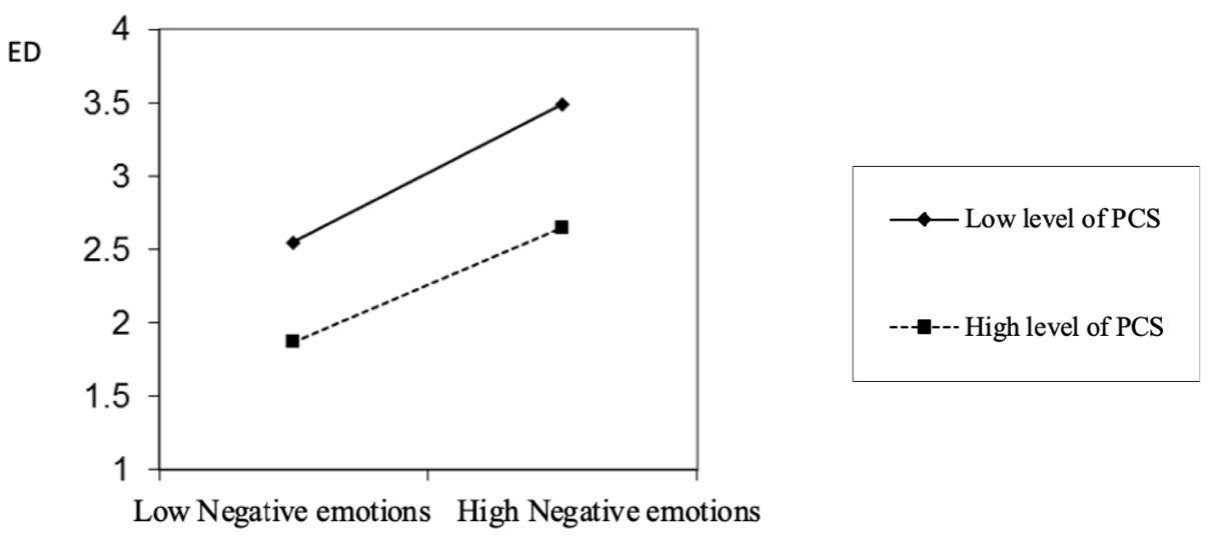
The moderating effect of PCS.

H7 posits that PCS exerts a negative moderating effect on the relationship between negative emotions and ego depletion. The verification process is as follows: First, as shown in Model 8 of Table 5, there is a significant positive correlation between negative emotions and ego depletion (*r* = 0.48, *p* < 0.01). Second, Model 10 of Table 5 indicates a significant negative correlation (*r* = −0.38, *p* < 0.01) between the main effect of PCS and ego depletion. Finally, the results from Model 12 in Table 5 demonstrate that as PCS increases, the relationship between negative emotions and ego depletion remains relatively unchanged (*r* = –0.04, *p* > 0.05). This indicates that the moderating effect of PCS is not significant. Therefore, H7 is not supported. The moderating effect of PCS is illustrated in Figure 3. There is no significant difference in the positive relationship between negative emotions and ego depletion when PCS is high vs. when it is low. This observation further confirms that H7 is not supported.

H8 posits that POS exerts a moderating role in the indirect effects of negative emotions on OCB and CWB through ego depletion. The data results are presented in Table 6. As shown in Table 6, when POS is high, the indirect effect of negative emotions on OCB through ego depletion is significant (*r* = –0.049, 95% CI=[-0.065,−0.008]); When POS is low, the indirect effect of negative emotions on OCB through ego depletion is significant [*r* = –0.053, 95% CI = (−0.130, −0.031)]. Hence, there is a significant difference between the estimated results of these two indirect effects [Δ = 0.004, 95% CI = (0.008, 0.090)], indicating that the indirect effect is more significant when POS is low. Therefore, H8a is supported. Additionally, as shown in Table 6, when POS is high, the indirect effect of negative emotions on CWB through ego depletion is significant [*r* = 0.041, 95% CI = (0.001, 0.056)]; When POS is low, the indirect effect of negative emotions on CWB through ego depletion is significant [*r* = 0.044, 95% CI = (0.003, 0.112)]. Thus, there is a significant difference between the estimated results of these two indirect effects [Δ = −0.003, 95% CI = (−0.076, −0.001)], indicating that the indirect effect is more significant when POS is low. Therefore, H8b is supported.

**Table 6:** The moderating mediation under the POS.

Variable	NM→**ED**→**OCB**		NM→**ED**→**CWB**	
	Indirect effect *r*	95% CI	Indirect effect *r*	95% CI
High level of POS	−0.049	(−0.065, −0.008)	0.041	(0.001, 0.056)
Low level of POS	−0.053	(−0.130, −0.031)	0.044	(0.003, 0.112)
Difference	0.004	(0.008, 0.090)	−0.003	(−0.076, −0.001)

H9 posits that PCS exerts a moderating role in the indirect effects of negative emotions on OCB and CWB through ego depletion. First, the verification result of H7 has already indicated that the moderating effect of PCS on the relationship between negative emotions and ego depletion is not significant. Furthermore, when PCS is set at high and low levels, respectively, the difference between the estimated results of the indirect effect of negative emotions on OCB through ego depletion is not significant. At the same time, the 95% confidence interval is [−0.015, 0.033], and 0 is obviously included in this interval. Therefore, this indicates that H9a is not supported. In addition, when PCS is set at high and low levels, respectively, the difference between the estimated results of the indirect effect of negative emotions on CWB through ego depletion is also not significant. At the same time, the 95% confidence interval is [−0.029, 0.013], and 0 is also included in this interval. Therefore, H9b is not supported.

The ceiling effect of Perceived Colleague Support (PCS) was tested through regression analysis. The steps are as follows: Descriptive test: PCS was measured by a 7-point scale with a mean of 3.88 and a standard deviation of 0.56. The data were concentrated in the high score segment, the standard deviation was much smaller than 1/4 of the full range, and the histogram showed a right-skewed distribution; High score segment proportion: We counted the proportion of participants with PCS scores between 6 and 7, and the result was ≥20%, which met the basic judgment standard; Quantitative indicators: We calculated the ceiling coefficient (CC) of PCS as 0.89 and the effective ceiling rate (ECR) as 35%, which was determined as a moderate ceiling effect; Reliability test: After deleting any high-score items of PCS, the Cronbach's α coefficient changed from 0.862 to 0.858 without significant improvement, verifying the existence of the ceiling effect; ? Conclusion: There is a moderate ceiling effect on perceived colleague support (PCS), which leads to the non-significant moderating effect. Due to the concentration of data in the high score segment, it is impossible to distinguish the real perceived differences of employees in perceived colleague support.

### General discussion

#### Conclusion and discussion

From the interaction perspective of organizational behavior, it is meaningful to understand both the overall impact of negative emotions on employee behaviors and the moderating roles among these individual factors. Based on the self-control perspective, this study, drawing on social exchange theory and mood congruence theory, finds that negative emotions exert a significant negative impact on OCB and a significant positive impact on CWB. Negative emotions have a significant positive impact on ego depletion, which plays a mediating role in the process of negative emotions influencing OCB and CWB. POS negatively moderates the relationship between negative emotions and ego depletion, whereas the moderating role of PCS in this relationship is not significant. POS exerts a moderating role in the indirect effects of negative emotions on OCB and CWB through ego depletion, whereas PCS does not. In general, Hypotheses 1, 2, 3, 4, 5, 6, 8a, and 8b of this study are supported, while Hypotheses 7, 9a, and 9b are not supported.

Although previous studies have also focused on the relationship between employee emotions and behaviors at work, most of these studies have only examined the correlation between the two variables, without investigating the consistency and continuity of the relationship between emotions and behaviors from a dynamic change perspective. This study collected employees' emotional and behavioral data through work diaries, which take into account the fluctuating characteristics of emotions and behaviors. Based on this, the research conclusions are more objective and practical. In addition, this study incorporates the differences in perception among individuals (POS and PCS) as higher-level variables, which enables the model to cover two levels: within-individual and between-individual, further enhancing the comprehensiveness of the research conclusions.

#### Discussion on mediating effect

The results of this study indicate that during daily fluctuations, negative emotions are negatively correlated with employees‘ OCB while being positively correlated with their CWB. This positive-negative relationship reveals the two extreme impacts of employees' daily negative emotions. The research model that examines the effects of negative emotions on both OCB and CWB not only broadens the research scope of psychological theories related to negative emotions but also holds important guiding significance for organizational behavior research on employee behaviors.

From the perspective of the core logic of the ego depletion theory, ego depletion refers to the temporary exhaustion of individual self-control resources after continuous self-regulation, which fundamentally affects the allocation of individual behavioral resources and behavioral decision-making. This mechanism exerts heterogeneous impacts on OCB and CWB due to their distinct behavioral natures. OCB, as an extra-role positive behavior not explicitly stipulated by the organization, does not have mandatory constraints, and its occurrence depends entirely on the individual's voluntary investment of cognitive resources, emotional energy, and motivational motivation—these investments are all based on sufficient self-control resources. When negative emotions induce ego depletion, individuals' self-control resources are significantly consumed, and they will spontaneously activate the “resource conservation” mechanism to avoid excessive consumption of limited resources in non-essential behaviors. At this point, the motivation and ability of individuals to actively engage in OCB are completely inhibited: negative emotions cannot directly affect OCB, but must exert an indirect impact through the full intermediary role of ego depletion, which leads to the complete mediating effect of ego depletion in the relationship between negative emotions and OCB.

In sharp contrast, CWB, as an extra-role negative behavior that harms organizational interests and interpersonal relationships, is essentially an impulsive expression of negative emotions, and its occurrence is more dependent on the decline of individual inhibitory control ability rather than active resource investment. According to the ego depletion theory, the decline of self-control resources caused by ego depletion will weaken individuals' ability to restrain impulsive negative behaviors, making it easier for negative emotions to be released in the form of CWB. However, unlike OCB, negative emotions themselves can directly trigger CWB through an emotional response pathway independent of ego depletion: strong negative emotions (such as anger, resentment, and dissatisfaction) can directly evoke individuals' retaliatory psychology and negative feedback tendencies, prompting them to adopt CWB to vent their emotions and respond to perceived unfairness or negative experiences. This means that negative emotions can affect CWB in two parallel paths—directly through emotional arousal, and indirectly through ego depletion reducing inhibitory control—thus leading to the partial mediating effect of ego depletion in the relationship between negative emotions and CWB.

The social exchange theory further supplements and deepens the interpretation of the above differential mediating mechanisms by revealing the relational logic behind OCB and CWB. The occurrence of OCB is based on the reciprocal social exchange relationship between individuals and organizations: individuals take the initiative to engage in OCB to maintain a positive exchange state, expecting to obtain potential long-term returns (such as organizational recognition, better interpersonal relationships, and career development space). Ego depletion undermines this reciprocal exchange process: when self-control resources are exhausted, individuals lose the motivation to maintain positive exchange, and their behavioral focus shifts from “relationship maintenance” to “resource conservation,” thus completely blocking any possible direct impact of negative emotions on OCB and strengthening the complete mediating role of ego depletion.

For CWB, it is a typical negative social exchange behavior that violates the principle of reciprocity: individuals perceive unfair exchange relationships (triggered by negative emotions) and respond with CWB to “balance” the exchange inequality. This negative exchange response is more intuitive and less resource-dependent than OCB—even without ego depletion, the perception of unfairness caused by negative emotions can directly induce CWB. Ego depletion only plays a role in amplifying this effect: the decline of self-control resources reduces individuals' ability to restrain negative exchange behaviors, making it more likely for negative emotions to be translated into CWB. The coexistence of the direct negative exchange path and the indirect ego depletion amplification path ultimately leads to the partial mediating effect of ego depletion, which also explains the fundamental difference between the two mediation modes.

In addition, the present study finds that ego depletion is positively correlated with employees' negative emotions, which is consistent with traditional research suggesting that external activities lead to the depletion of limited self-control resources. The negative emotions in the present study can be understood as a special type of external impact, which is an unavoidable negative affective state experienced by employees in their daily work, due to factors such as interpersonal conflicts and the external environment. Existing studies have experimentally demonstrated that positive emotions have a preventive and compensatory effect on ego depletion (Tice et al., 2007). In contrast, the results of the present study indicate that negative emotions lead to greater ego depletion. This not only confirms the conclusions of previous studies from an opposite perspective but also extends the existing research.

#### Discussion on moderated mediating effect

The study finds that POS, a variable reflecting between-individual differences, can negatively moderate the positive relationship between negative emotions and ego depletion. Specifically, when an employee's POS is enhanced, the positive relationship between negative emotions and ego depletion is weakened. This finding is consistent with previous studies that have demonstrated the impact of POS on employee behaviors through the mediation of psychological capital (Yang et al., 2020). Like POS, PCS is an important component of social exchange theory (Eisenberger et al., 1986). However, the present study finds that neither the moderating effect nor the moderated mediating effect of PCS is significant. We argue that this result is inevitably influenced by China's national conditions and organizational environment, as all data in the present study are collected from Chinese organizational contexts. First, China is a country that advocates collectivism, and the concept of collectivism is deeply rooted in society, while individualism is not widely recognized (Hofstede, 1984; Minkov and Kaasa, 2022). This explains why POS plays a significant role, whereas PCS does not. Under the influence of collectivism, POS has a greater impact on employees, which is more capable of changing their psychological states and thus more effectively mitigates the effect of negative emotions on ego depletion. Second, studies have shown that Asian countries have a higher power distance compared to Western countries (Hofstede, 1984). In cultural environments with high power distance, the level of trust in others is relatively low (Thanetsunthorn and Wuthisatian, 2019). In the workplace, this is reflected in the low level of trust among coworkers. Third, combined with the results of empirical tests, Perceived Colleague Support (PCS) exhibits a moderate ceiling effect, with data concentrated in the high-score segment, which makes it impossible to distinguish the real differences in employees' perceptions of colleague support. This may lead to the non-significant moderated mediation mechanism of PCS. However, there may be other possibilities for the non-significant moderated mediation effect of PCS. In addition to cultural factors, the failure of perceived colleague support (PCS) to play a moderating role may also be related to the potential ceiling effect and the time mismatch in measurement timing. The potential cap effect of PCS scores. This study used a 7-point Likert scale to measure PCS, with an average score of 3.88 and a standard deviation of 0.56. The small standard deviation (less than 1.0) as well as the mean close to the scale median (4.0) may indicate a limited range of variation in PCS scores. This limited variability would prevent PCS from showing enough variation across individuals to make it difficult to moderate the relationship between negative emotions, self-exhaustion, and employee behavior, despite its theoretical buffering effect. The problem of mismatch between measurement timing and chronological order. PCS, which is an interindividual variable, was measured only once at the baseline survey and reflects an individual's stable perception of support from colleagues. In contrast, negative emotions and ego depletion, as intra-individual variables, are collected through diary studies and present daily fluctuating changes. This mismatch in time order results in the inability of the stable PCS to capture the dynamic daily variation of the core variables, weakening its moderating effect and ultimately leading to the inefficiency effect. Therefore, we consider this another important reason for the non-significant moderating effect of PCS.

### Theoretical contributions

This study employs the self-control theory as the mediating mechanism to investigate the relationship between negative emotions and employee behaviors. Based on the social exchange theory (Eisenberger et al., 1986), it examines the impact of employees' individual perceptions at work on this psychological mechanism. The results indicate that the present study holds strong theoretical significance, which is specifically reflected in the following aspects:

First, when exploring the relationship between negative emotions and employee behaviors, this study subdivides employee behaviors into opposing OCB and CWB. Compared with most previous studies that only focused on the relationship between negative emotions and OCB or between negative emotions and CWB individually, this study incorporates these two opposing behaviors into the research model simultaneously. This makes the research on the relationship between emotions and behaviors more comprehensive and significantly contributes to the theoretical enrichment of the relevant literature on negative emotions, OCB, and CWB.

Second, the model of this study introduces ego depletion as a mediating variable between negative emotions and two types of employee behaviors (OCB and CWB). The data results confirm that ego depletion plays a mediating role in the impact of negative emotions on employee behaviors. The introduction of ego depletion in the present study enriches the literature on ego depletion theory. Furthermore, it expands the application scope of ego depletion theory, particularly in the Chinese cultural context. Since its proposal, ego depletion theory has attracted the attention and research of scholars in fields such as psychology and organizational behavior. This study examines ego depletion as a mediating variable to investigate the relationships between negative emotions and OCB, as well as CWB, thereby integrating ego depletion theory with psychology and organizational behavior. This integration makes the theoretical literature on emotions and ego depletion more comprehensive and substantial.

Thirdly, research on POS based on social exchange theory has increasingly attracted the attention of scholars in the fields of organizational behavior and human resource management. The model of this study treats POS as a between-individual level variable, breaking the convention of traditional studies that uniformly conduct single-level research on organizational support and employee behaviors. This holds in-depth significance for improving the application of social exchange theory in Chinese organizational contexts and further enriches the theoretical literature on POS. In addition, the research result that POS exhibits a moderated mediating effect is conducive to promoting studies on the mechanism of the ego depletion theory. In addition, it also holds significant implications for the integrated research of social exchange theory and ego depletion theory.

Finally, in recent years, the ego depletion theory has encountered a replication crisis—some studies have failed to replicate the classic ego depletion effect, thereby triggering debates about the validity of the limited resource model (Baumeister et al., 2018). On the one hand, the result of this study showing that daily negative emotions predict increased ego depletion is consistent with the core proposition of the limited resource model (self-control resources are consumed by negative emotional regulation); on the other hand, this study points out that the motivational interpretation of ego depletion (Inzlicht and Schmeichel, 2012)—which argues that ego depletion reflects a shift in motivation rather than the actual exhaustion of resources—is not mutually exclusive with the findings of this study, because negative emotions may also trigger motivational changes, manifested as a reduction in self-control capacity. In addition, the diary study design adopted in this study (by recording the daily changes of individuals' ego depletion) helps to shed light on these ongoing theoretical debates: unlike cross-sectional designs that only measure stable trait-like ego depletion, daily measurement can capture the dynamic changes of self-control capacity, thereby providing more ecologically valid evidence to distinguish between resource-based and motivational interpretations.

### Practical implication

Since this study focuses on the impact of negative emotions on OCB and CWB, and these three variables occur relatively frequently in employees' work practices, the research results of this study also have typical practical implications, which are specifically reflected in the following aspects:

First, this study suggests that the negative emotions employees encounter at work can inhibit the occurrence of their extra-role behaviors, which are beneficial to organizational development, such as proactively helping coworkers and actively proposing suggestions for organizational improvement. In contrast, these negative emotions will promote the occurrence of adverse behaviors that harm organizational development. Therefore, employees‘ negative emotions are detrimental to the organization's survival and development. Based on the above conclusions and discussions, one of the practical implications of this study is to help organizations and managers gain a more precise and intuitive understanding of the detrimental impact of employees' negative emotions on the organization, thereby further enhancing managers‘ awareness of employees' negative emotions. Specifically, in the actual management, it requires managers to create a more positive cultural atmosphere for the enterprise from various aspects, reduce the occurrence of negative emotions among employees at work, and thus alleviate the negative impact that hinders organizational development.

Second, this study finds that ego depletion plays a significant mediating role in the impact of negative emotions on employee behaviors, indicating that the individual's self-control resources have a substantial impact on their psychological and behavioral states. This suggests that in practical management practices, organizations and managers should minimize the depletion of employees' self-control resources, especially the depletion caused by external activities. Since self-control resources can be replenished within a certain period, organizations and managers should also provide employees who have experienced ego depletion with adequate rest time to help them recover self-control resources more quickly, thereby effectively mitigating the negative effects caused by ego depletion.

Finally, this study finds that POS can negatively moderate the positive impact of negative emotions on ego depletion, indicating that POS can play a positive role in mitigating the negative effects caused by negative emotions. Consequently, organizations will also benefit from such positive support. This has significant implications for management practice. In actual management, most organizations attach importance to technical training and salary incentives, but often overlook the importance of POS, especially in providing emotional support for employees. Practices such as recognition, positive feedback, and care for employees‘ personal lives are all conducive to enhancing employees' POS, fostering a sense of confidence and an optimistic psychological state, and thereby strengthening their work motivation. Therefore, organizations and managers should provide employees with organizational support in various aspects throughout the management process. For example, helping employees with difficulties and taking care of their welfare. In summary, enterprises should adopt specific policies and measures to enhance the employee POS, enabling it to fully promote the development of both employees and the organization.

## Limitations and future research

This study has several limitations that provide valuable avenues for future research.

First, the sample was restricted to 155 employees from nine enterprises in Wuhan, China, with a skewed industry distribution (primarily in the administrative and marketing sectors) and a predominantly high level of educational attainment. Consequently, the generalizability of the research conclusions is somewhat limited in terms of regional and occupational scope. Future research should expand the sample size, overcome geographical and industrial limitations (such as including the manufacturing and healthcare sectors), and select employees from more diverse backgrounds to further verify the broad applicability of these findings.

Second, this study included only two within-individual control variables: weather and outdoor working hours. To avoid participant fatigue and maintain data quality inherent in the daily diary method, additional relevant control variables could not be fully incorporated, which may introduce unmeasured confounding effects. Future studies should optimize the data collection design to systematically include more comprehensive control variables, thereby further excluding potential confounding factors and improving the reliability of the results.

Third, China's unique cultural context, characterized by high power distance and collectivism, may significantly affect the relationships among the core variables, potentially contributing to the non-significant moderating effect of perceived coworker support (PCS). In this high-power-distance context, employees may rely more heavily on formal organizational support rather than informal peer support, thereby weakening the buffering effect of PCS on negative emotions and ego depletion. While Western research often highlights the significant moderating role of PCS, our findings suggest that cultural nuances may alter this dynamic. Future research should utilize heterogeneous, cross-cultural samples to explore the boundary conditions of these moderating effects.

Finally, regarding the analytical approach, this study utilized Hierarchical Linear Modeling (HLM) to test the cross-level moderated mediation hypotheses. While HLM is a robust and well-established tool for examining relationships across nested levels and testing “slopes-as-outcomes” interactions, it does not permit the evaluation of the global structural model fit in the way that Multilevel Structural Equation Modeling (MSEM) does. MSEM is highly advantageous for simultaneously accounting for measurement error and providing global fit indices for the entire structural model. However, estimating an MSEM with multiple latent variables and cross-level interactions requires a substantially larger Level-2 sample size to ensure model convergence and stable parameter estimates. Given our sample of 155 participants at the between-individual level, HLM was deemed the most appropriate and rigorous choice to avoid over-parameterization. Future research utilizing larger multi-level samples should employ MSEM to further validate the overall structural fit of this theoretical model.

## Data Availability

The original contributions presented in the study are included in the article/supplementary material, further inquiries can be directed to the corresponding authors.

## Footnotes

1 Ethical Approval Statement: Ethical review and approval were not required for the study on human participants in accordance with the local legislation and institutional requirements. Written informed consent from the participants was not required to participate in this study in accordance with the national legislation and the institutional requirements.
